# Stimulation of Chitin Synthesis Rescues *Candida albicans* from Echinocandins

**DOI:** 10.1371/journal.ppat.1000040

**Published:** 2008-04-04

**Authors:** Louise A. Walker, Carol A. Munro, Irene de Bruijn, Megan D. Lenardon, Alastair McKinnon, Neil A. R. Gow

**Affiliations:** School of Medical Sciences, Institute of Medical Sciences, University of Aberdeen, Foresterhill, Aberdeen, United Kingdom; Johns Hopkins University School of Medicine, United States of America

## Abstract

Echinocandins are a new generation of novel antifungal agent that inhibit cell wall β(1,3)-glucan synthesis and are normally cidal for the human pathogen *Candida albicans*. Treatment of *C. albicans* with low levels of echinocandins stimulated chitin synthase (*CHS*) gene expression, increased Chs activity, elevated chitin content and reduced efficacy of these drugs. Elevation of chitin synthesis was mediated via the PKC, HOG, and Ca^2+^-calcineurin signalling pathways. Stimulation of Chs2p and Chs8p by activators of these pathways enabled cells to survive otherwise lethal concentrations of echinocandins, even in the absence of Chs3p and the normally essential Chs1p, which synthesize the chitinous septal ring and primary septum of the fungus. Under such conditions, a novel proximally offset septum was synthesized that restored the capacity for cell division, sustained the viability of the cell, and abrogated morphological and growth defects associated with echinocandin treatment and the *chs* mutations. These findings anticipate potential resistance mechanisms to echinocandins. However, echinocandins and chitin synthase inhibitors synergized strongly, highlighting the potential for combination therapies with greatly enhanced cidal activity.

## Author Summary

Fungal pathogens are increasingly important agents of human disease and are also difficult to treat since few antifungal agents kill the invading organism. The cell wall of a fungus is essential for its viability and this can be attacked by a new generation of antifungal antibiotics called echinocandins. Echinocandins such as caspofungin are normally cidal for the human pathogen *Candida albicans*. These inhibit the synthesis of *β*(1,3)-glucan, a major strength-imparting polysaccharide in the cell wall. Treatment of *C. albicans* with echinocandins *in vitro* stimulated the formation of a second cell wall polysaccharide—chitin, which rescued the cells. Treatments that increased the chitin content of the *C. albicans* cell wall reduced the efficacy of echinocandins and could even induce the formation of novel structures such as a salvage septum that enabled the cells to continue to undergo cell division under otherwise lethal conditions. Combined treatments with echinocandins and chitin synthase inhibitors synergized strongly, highlighting the potential for potent combination therapies with enhanced fungicidal activity.

## Introduction

In fungi, two covalently cross-linked polysaccharides, β(1,3)-glucan and chitin, form a primary scaffold that is responsible for structural integrity and shape of the cell wall [1]–[4]. Other β-linked polysaccharides and glycosylated proteins are attached to this glucan-chitin core, thus modifying the properties of the wall. The integrity of the cell wall scaffold must, however, be monitored and regulated constantly to ensure cell viability. This is not a trivial challenge since surface expansion during growth and cellular morphogenesis requires a delicate balance to be maintained between the rigidity and the flexibility of the cell wall. The cell wall must be able to expand under the outwardly directed and variable force of cell turgor, whilst maintaining sufficient rigidity to prevent cell lysis. This balance between plasticity and rigidification must also be achievable in the presence of extrinsic factors such as inhibitory molecules and enzymes in the environment that may attack the integrity of the cell wall. Responses to cell wall damage involve a sophisticated homeostatic mechanism that is mediated via a signalling network which communicates information about physical stresses at the cell surface to the biosynthetic enzymes that orchestrate cell wall synthesis and repair. The signalling pathways and transcription factors that mediate this repair response are termed the cell wall salvage or cell wall compensatory mechanisms [5]–[8].

Echinocandins are a new class of antifungal agent, which are non-competitive inhibitors of β(1,3)-glucan synthase [9]. Caspofungin is the first echinocandin to be approved for clinical use and is fungicidal for *Candida albicans*, and other *Candida* species, and fungistatic for *Aspergillus fumigatus* [10],[11]. It is active against isolates of *Candida* spp. that are resistant to other antifungals such as fluconazole [12]. Deletion of both copies of the *FKS1* gene is lethal in *C. albicans*, although point mutations in *FKS1* can arise that result in reduced susceptibility to caspofungin [9], [13]–[15]. *FKS1* point mutations associated with resistance accumulate in two hot spot regions that encode residues 641–649 and 1345–1365 of *Ca*Fks1p in *C. albicans* and other species [14]–[17]. Fungi that are inherently less susceptible to echinocandins, have a tyrosine at residue 641 compared to phenylalanine in that position in *Ca*Fks1p [16],[18], suggesting sequence divergence around the hot spot regions may contribute to reduced echinocandin susceptibility.

In *Saccharomyces cerevisiae* deletion of *ScFKS1* is not lethal and inhibition of β(1,3)-glucan synthesis or damage to β(1,3)-glucan results in increased levels of chitin synthesized by *Sc*Chs3p [7],[19]. *Scchs3*Δ mutants are hypersensitive to caspofungin [20] and *ScCHS3* and *ScFKS1* are synthetically lethal [21],[22] suggesting that *Scfks1*Δ mutants depend on chitin synthesis for their survival. In addition microarray analyses have shown that *CaCHS2* expression increases in response to caspofungin treatment [23],[24]. Treatment of *S. cerevisiae* [25] and *Cryptococcus neoformans* [26] with caspofungin results in activation of the PKC cell integrity pathway via phosphorylation of the mitogen activated protein kinase, *Sc*Slt2p/*Sc*Mkc1p. *C. albicans MKC1* expression has been found to increase in response to caspofungin treatment [27] and deletants in *ScSLT2* are hypersensitive to caspofungin [20],[25]. Damage to the cell wall involves cell wall protein sensors which transmit signals that lead to activation of the *Sc*Rho1p GTPase, which activates β(1,3)-glucan synthase as well as *Sc*Pkc1p and hence the PKC cell integrity MAP kinase cascade [8]. The downstream target of this cell wall salvage pathway is the *Sc*Rlm1p transcription factor, which activates transcription of cell wall related genes [28].

The Ca^2+^-calcineurin pathway has also been implicated in the regulation of cell wall biosynthesis [29]–[31]. *C. albicans* calcineurin mutants are hypersensitive to caspofungin, suggesting that the calcineurin pathway is involved in the response to cell wall damage caused by caspofungin [32]. Combined treatment with caspofungin and the calcineurin inhibitor, cyclosporin A, prevents the paradoxical effect of increased survival that is sometimes seen at echinocandin concentrations well above the typical minimal inhibitory concentration (MIC) [27]. The calcineurin inhibitors, FK506 and cyclosporin A, have also been shown to act synergistically with caspofungin against *Aspergillus fumigatus* and *Cryptococcus neoformans* [26], [33]–[35].

A primary response of fungi to cell wall damage is to up-regulate chitin synthesis. In *C. albicans* there are four chitin synthase enzymes, *Ca*Chs1p, *Ca*Chs2p, *Ca*Chs3p and *Ca*Chs8p. *Ca*Chs1p is an essential class II enzyme that synthesizes the chitin of the primary septum and contributes to lateral wall integrity [36]; *Ca*Chs2p and *Ca*Chs8p are two class I enzymes that account for almost all measured chitin synthase activity *in vitro* [37]–[39] and *Ca*Chs3p is a class IV enzyme that synthesizes the majority of cell wall chitin, including the septal chitin ring [40]. While *Ca*Chs3p is predominantly regulated at the post-transcriptional level, *CaCHS1*, *CaCHS2*, and *CaCHS8* can all be transcriptionally activated in response to stimulants of the PKC, Ca^2+^-calcineurin and HOG signalling pathways [31]. Here we show that pre-treatment of cells with activators of these pathways activates *CaCHS* transcription and leads to the selection of cells with increased cell wall chitin that survive otherwise lethal concentrations of caspofungin. We also show that activation of the cell wall compensatory pathways can induce the synthesis of a novel salvage septum even in the absence of *Ca*Chs3p and *Ca*Chs1p which are normally required for septum formation and viability. Rescue of such cells was strictly dependent on chitin synthesis from residual class I enzymes and combinations of echinocandins and chitin synthase inhibitors exhibited synergy in the killing of *C. albicans*.

## Results

### Echinocandins induce CHS expression via three signalling pathways

**Figure 1:**
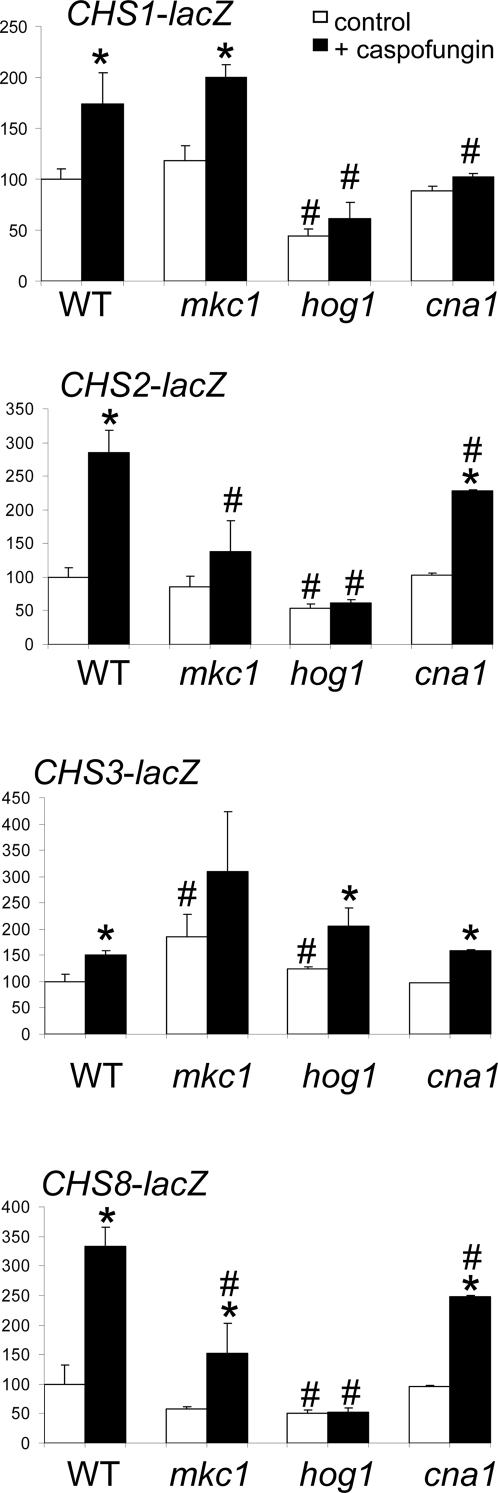
Upregulation of *CHS* expression in response to caspofungin is dependent on the PKC, HOG and Ca^2+^-calcineurin pathways. Response of *CHS* promoter-*lacZ* reporters in the absence (empty bars) and presence (solid bars) of caspofungin in signal transduction mutants. Error bars are S.D. (n = 9 from three separate experiments). Asterisks indicate significant differences (*p*<0.05) compared to the untreated control in the same genetic background. # indicates significantly different (*p*<0.05) to the wild type cells in the same growth conditions. The fold inductions for LacZ activity upon caspofungin exposure are shown in Table S2.

To test whether exposure to echinocandins induced chitin synthesis we first used a *lacZ*-reporter system to measure the response of the four *C. albicans CHS* promoters to echinocandins at concentrations below their MICs. Caspofungin (Figure 1) and echinocandin B, cilofungin and anidulafungin (data not shown) activated expression of *CHS1*, *CHS2* and *CHS8*. The level of expression of the class IV *CHS3* was only increased significantly with anidulafungin (data not shown). Previously we showed that the PKC, Ca^2+^-calcineurin and HOG pathways all regulated *CHS* expression [31]. We then used reporter constructs to establish which signalling pathways were required to activate these transcriptional responses to echinocandins. The mutants tested had the following genes deleted; *HOG1* encoding the MAP kinase of the HOG pathway, *MKC1* encoding the MAP kinase of the PKC pathway, and the calcineurin catalytic subunit *CNA1*. Mutations in these pathways affected both the basal level of gene expression and the response to caspofungin. Mutant strains with deletions in the HOG pathway (*hog1*Δ) showed no increase in expression of *CHS1*, *CHS2* and *CHS8* when caspofungin was applied (Figure 1). Up-regulation of *CHS1* was not seen in *cna1*Δ mutants after caspofungin addition, therefore the Ca^2+^-calcineurin pathway was involved in the regulation of *CHS1*. Equivalent analyses showed that the PKC pathway contributed to the up-regulation of the expression of *CHS2* and *CHS8* upon exposure to caspofungin.

**Figure 2:**
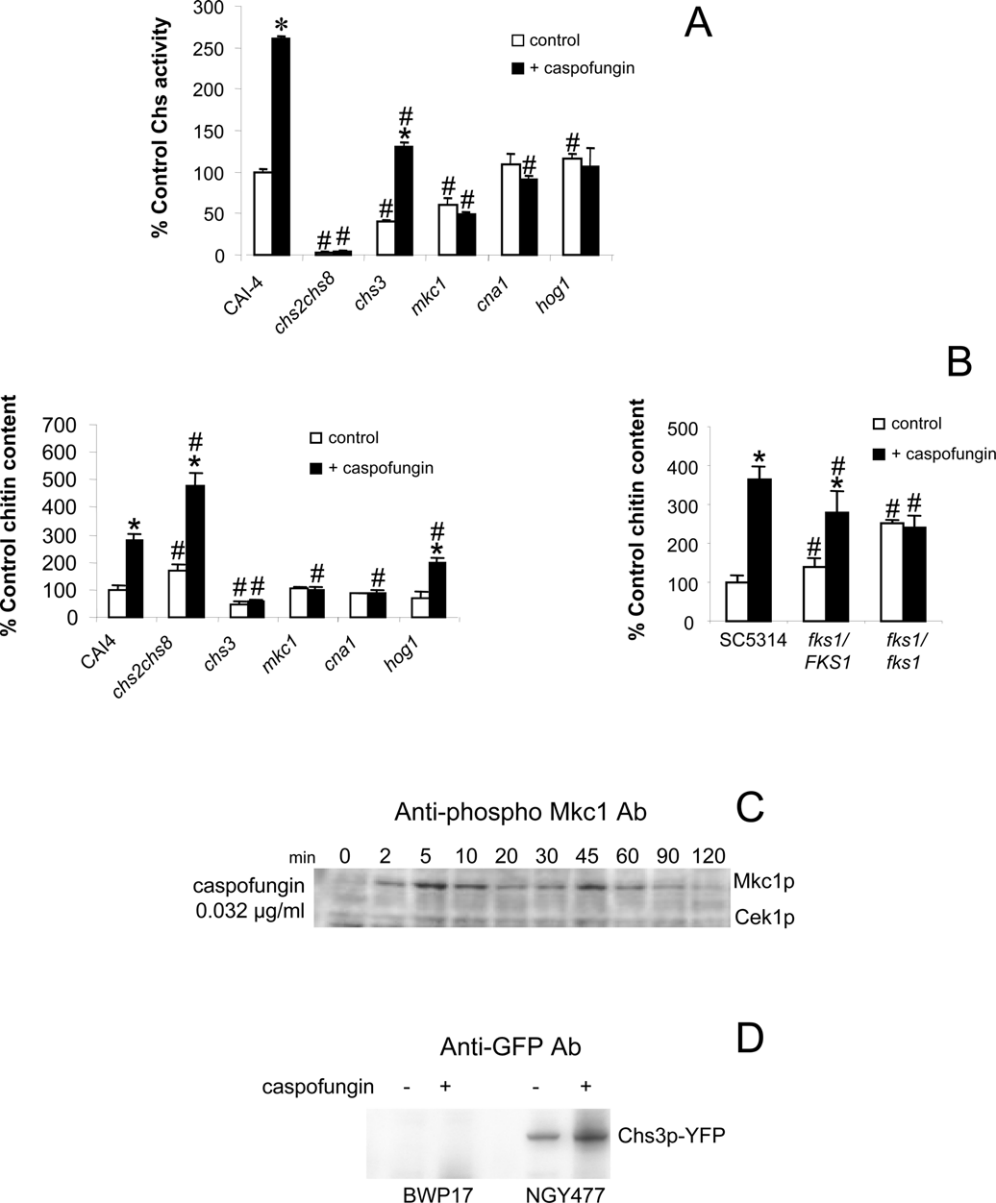
Activation of chitin synthesis by caspofungin. Chitin synthase activity (A) and chitin content (B) of various chitin synthase, signal transduction and *fks1* point mutants (*fks1*/*FKS1* = NR2; *fks1*/*fks1* = NR3) in the absence (empty bars) and presence (solid bars) of 0.032 µg/ml caspofungin. Isolation of NR2 and NR3 are described in Douglas et al (1997) [9]. Asterisks indicate significant differences (*p*<0.05) compared to the untreated control for each strain. # indicates a significant difference (*p*<0.05) to the wild type cells in the same growth conditions. Chitin synthase assays were performed in triplicate (average ±SD, n = 3). Cell wall chitin assays were performed five times on three biologically independent samples (average ±SD, n = 15). Time course (min) of Mkc1 phosphorylation in wild type cells in response to 0.032 µg/ml caspofungin (C). Western analysis of Chs3p levels in cells exposed to 0.032 µg/ml caspofungin, strain NGY477 carries Chs3p C-terminally tagged with YFP and BWP17 is the untagged parental strain (Table S1) (D).

Caspofungin treatment of cells also led to a 2.5-fold increase in specific chitin synthase activity measured in mixed membrane preparations (Figure 2A), and a near 3-fold increase in the chitin content of the cells (Figure 2B). The measured stimulation of chitin synthase activity was dependent on the presence of two class I enzymes Chs2p and Chs8p and on the Ca^2+^-calcineurin, PKC and HOG pathways (Figure 2A). The total chitin content stimulated by caspofungin was largely dependent on Chs3p and this also required a functional PKC pathway and the presence of calcineurin (Figure 2B). The HOG pathway also had a significant influence on the stimulation of chitin content by caspofungin. The effect of point mutations in *FKS1* was also determined by measuring cell wall chitin content (Figure 2B). Strain NR3, which was resistant to caspofungin as a result of homozygous point mutations in the β(1,3)-glucan synthase gene *FKS1* [9] had an almost three-fold increase in chitin content and lost the stimulation of chitin synthesis by caspofungin (Figure 2B). We further implicated the PKC pathway in the response to caspofungin by showing phosphorylation of Mkc1p in wild type cells treated with caspofungin (Figure 2C). We also quantified an increase in Chs3p upon caspofungin treatment by western analysis using anti-GFP antibodies and a strain engineered with a C-terminal YFP tag fused to Chs3p [41] (Figure 2D). Therefore, the HOG, PKC and Ca^2+^-calcineurin signalling pathways were found to mediate the elevation of chitin synthase gene expression, chitin synthase activity and chitin content in response to caspofungin.

### Activation of cell wall salvage pathways protects against echinocandin treatment

**Figure 3:**
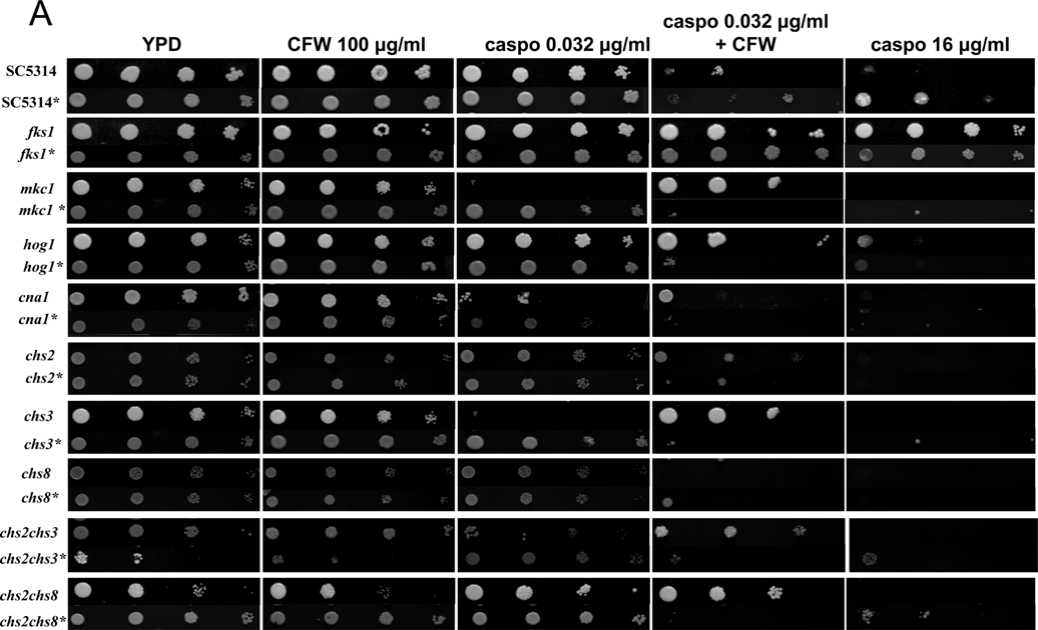
Pre-growing cells in CaCl_2_ and CFW reduces their susceptibility to caspofungin. Plate dilution sensitivity tests of wild type (SC5314), a *fks1* homozygous point mutant (NR3) and a range of signalling and chitin synthase single and double mutants on YPD agar containing CFW, caspofungin or CFW and caspofungin. Rows marked with * indicate pre-growth of the inoculum in YPD containing both CaCl_2_ and CFW to raise the chitin content of the cells. Cell numbers per spot are from 5000, 500, 50 to 5 cells, from left to right.

Having shown previously that Calcofluor White (CFW) and Ca^2+^ are activators of the cell wall compensatory signalling pathways that could stimulate chitin synthesis [31]; we determined whether pre-treatment of cells with such agonists could protect cells from the cidal affects of caspofungin. Inocula of wild type or various mutant strains of *C. albicans* were grown in YPD, with and without added CaCl_2_ and CFW, before washing, dilution and plating onto agar containing caspofungin and other supplements (Figure 3). The homozygous *fks1* point mutant, strain NR3, which was greatly reduced in sensitivity to caspofungin [9],[14] was included as a control. Under normal growth conditions, the *cna1*Δ, *mkc1*Δ and *chs3*Δ mutants were hypersensitive to a low concentration of caspofungin (0.032 µg/ml) compared to wild type cells. The strains did not show significant sensitivity to 100 µg/ml CFW alone but CFW was found to act synergistically with 0.032 µg/ml caspofungin to enhance killing (Figure 3). Only the *fks1* point mutant was able to grow at a higher caspofungin concentration (16 µg/ml). Pre-treatment of the inoculum by growth in CaCl_2_ and CFW (rows marked with asterisks) significantly reduced the efficacy of 16 µg/ml caspofungin against wild type cells and was dependent upon *MKC1*, *HOG1*, *CNA1*, and *CHS2*, *CHS3* and *CHS8* (Figure 3). At lower caspofungin concentrations less dependency on these genes was found. These results suggest that stimulation of chitin synthesis accounted for decreased caspofungin sensitivity and inhibition of chitin assembly increased caspofungin toxicity. Combining FK506 with caspofungin phenocopied the effects of the *cna1*Δ mutation (data not shown). Experiments were also carried out using CaCl_2_ or CFW pre-treatments alone. Priming cells with CaCl_2_ alone conferred more caspofungin protection than treatment with CFW alone (data not shown).

**Figure 4:**
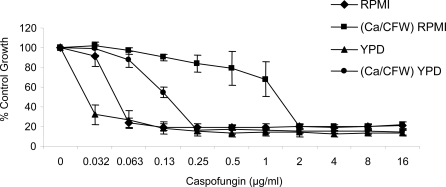
Pre-growing cells in CaCl_2_ and CFW increases their caspofungin MIC. The MIC of *C. albicans* strain SC5314 was measured in RPMI 1640 and YPD medium supplemented with caspofungin. The effect of growing the inoculum on YPD or YPD with CaCl_2_ and CFW was tested; cells pre-grown in CaCl_2_ and CFW had an increased MIC for caspofungin.

Pre-growing cells in CaCl_2_ and CFW supplemented medium was also found to protect cells against caspofungin in liquid culture on YPD or RPMI 1640. Using the CLSI method we determined that pre-growing cells with CaCl_2_ and CFW significantly increased the MIC for caspofungin by up to 6 doubling dilutions (Figure 4). The MIC to anidulafungin and micafungin was also increased by pre-treatment of wild type strains with CaCl_2_ and CFW however, MIC to fluconazole, amphotericin B, terbinafine and 5-flucytosine remained unchanged (data not shown).

**Figure 5:**
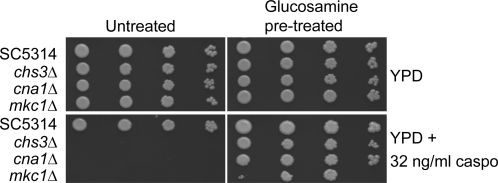
Cells pre-grown in glucosamine have reduced susceptibility to caspofungin. Pre-growing strains that were hypersensitive to caspofungin (*chs3*Δ, *cna1*Δ, *mkc1*Δ) in YPD plus 23 mM glucosamine reduced their susceptibility to 0.032 µg/ml caspofungin. Cell numbers per spot are from 5000, 500, 50 to 5 cells, from left to right.

Growth of *S. cerevisiae* on glucosamine–supplemented medium has been shown to stimulate chitin synthesis [42]. Therefore, *C. albicans* yeast cells were pre-grown on glucosamine-supplemented YPD to establish whether this would also lead to protection against caspofungin. Cells grown in YPD supplemented with 23 mM glucosamine had an almost two-fold increase in chitin content compared to the control cells (data not shown) and glucosamine-grown cells were considerably less sensitive to caspofungin (Figure 5). This protection did not require Chs3p, Cna1p and Mkc1p. Therefore, this compensatory mechanism could occur even in mutants with deletions in individual signalling pathways of the cell wall compensatory response and in the absence of Chs3p - the chitin synthase responsible for synthesizing the majority of chitin in wild type cells.

### Cells with elevated chitin content have reduced sensitivity to caspofungin

**Figure 6:**
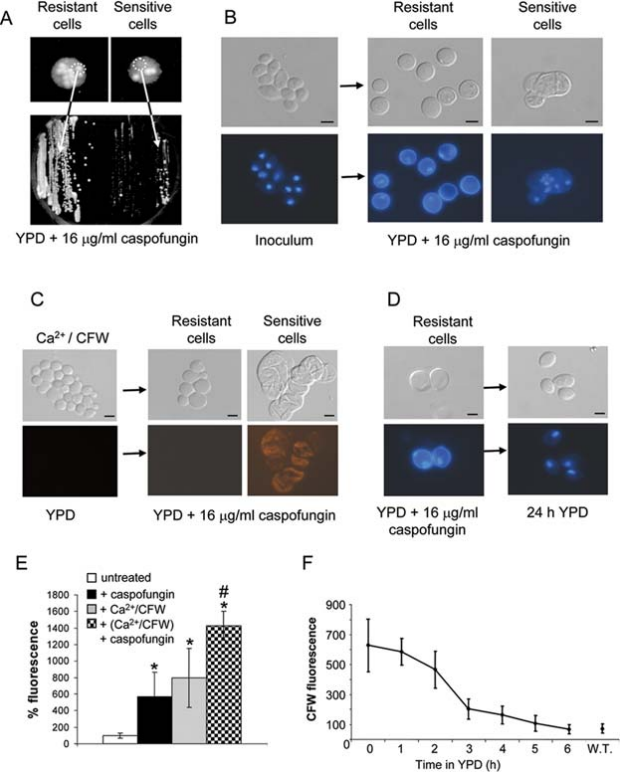
Transient elevation of chitin in cells with reduced susceptibility to caspofungin. CaCl_2_ and CFW pre-treated cells grown on medium containing 16 µg/ml caspofungin formed rich zones of growth within a lawn of surrounding cells (A, top panel). Inocula from the richer zones of the colony contain resistant cells that grew well on 16 µg/ml caspofungin (A, left hand panel), while the surrounding cells grow poorly to give few resistant colonies (A, right hand panel). DIC images and fluorescence micrographs of CFW and DAPI-stained cells (B, D) and propidium iodide stained cells (C). In (B) cells from the 16 µg/ml caspofungin-resistant, richer zones of the colony have a higher chitin content, i.e. stain more strongly with CFW, than caspofungin-sensitive cells and the inoculum. Caspofungin-sensitive cells are non-viable and take up propidium iodide whereas caspofungin-insensitive cells are viable and do not (C). Increased CFW staining of resistant cells was lost upon sub-culture under non-selective conditions, in the absence of caspofungin (D). The average relative chitin content of yeast cells, measured by intensity of CFW fluorescence, is shown for cells in untreated controls and after pretreatment with 0.32 µg/ml caspofungin or CaCl_2_ and CFW and for cells pre-treated with CaCl_2_ and CFW then grown in 0.32 µg/ml caspofungin (E). In (E) statistical differences are shown compared to untreated control (*P<0.001) and compared to Ca/CFW pre-treated cultures (# P<0.001) (by t-test). Under-non-selective conditions, the relative chitin content of resistant cells decreased progressively to that of the wild type (W.T.) as shown by the loss of CFW fluorescence (F). Error bars are S.D. (n = 6 from 3 separate experiments). Scale bars are 2 µm.

Wild type cells were grown in media containing CaCl_2_ and CFW, and then washed, diluted and plated onto YPD agar containing 16 µg/ml caspofungin. Colonies emerged that contained punctate rich zones of growth within a lawn of cells that when re-grown were resistant to 16 µg/ml caspofungin (Figure 6A, left panel). In contrast to the inoculum these resistant cells stained brightly with CFW indicating higher chitin content (Figure 6B) and they excluded the vital dye propidium iodide indicating they were viable (Figure 6C). In contrast, sensitive cells surrounding these rich zones of growth were susceptible to caspofungin, stained poorly with CFW, and were propidium iodide-sensitive and non-viable (Figure 6B and 6C). However, when inocula taken from parts of the colony outside the rich zones of growth were plated onto caspofungin-agar a few colonies arose which contained cells that were caspofungin insensitive and of high chitin content (Figure 6A, right panel). All colonies emerging on such plates could be propagated indefinitely on caspofungin-containing agar. When such cells were grown without caspofungin selection, they reverted quickly to become caspofungin-sensitive, and the reverted cells stained poorly with CFW (Figure 6D). Pre-treatment with CaCl_2_ and CFW stimulated the emergence of resistant colonies at a higher rate than occurred when cells were pre-treated with sub-MIC levels of caspofungin. For example, when cells were pre-treated with CaCl_2_ and CFW the rate at which resistant colonies emerged was approximately 1 in 120 cells. This compared to the emergence of resistant colonies from approximately 1 in 1.3×10^6^ cells that were pre-treated with 0.032 µg/ml caspofungin.

The intensity of CFW fluorescence of yeast cells was found to be an accurate reflection of the relative chitin content. Within a population of cells the average chitin content was found to be stimulated by treatment with CaCl_2_ + CFW or by sub-MIC concentrations of caspofungin (Figure 6E). Cells that were pre-treated with CaCl_2_ and CFW and then cultured in sub MIC concentrations of caspofungin had the highest levels of chitin (Figure 6E, and Figure S1). Thus exposure to CaCl_2_ and CFW and to caspofungin led to both an increase in the average chitin content of cells (Figure S1) and the selection of a sub-population of caspofungin resistant cells that formed zones of rich growth within colonies. When chitin-rich, caspofungin-insensitive cells were transferred to fresh YPD medium lacking caspofungin their chitin content declined to control unstimulated levels within 4–5 h equivalent to approximately 3–4 generations (Figure 6F). Thus the activation of chitin in response to caspofungin was a transient adaptation and upon removal of the drug chitin content returned to wild type levels.

### Salvage chitin synthesis involves multiple CHS isoenzymes and can generate a novel septum that rescues cell division and viability

**Figure 7:**
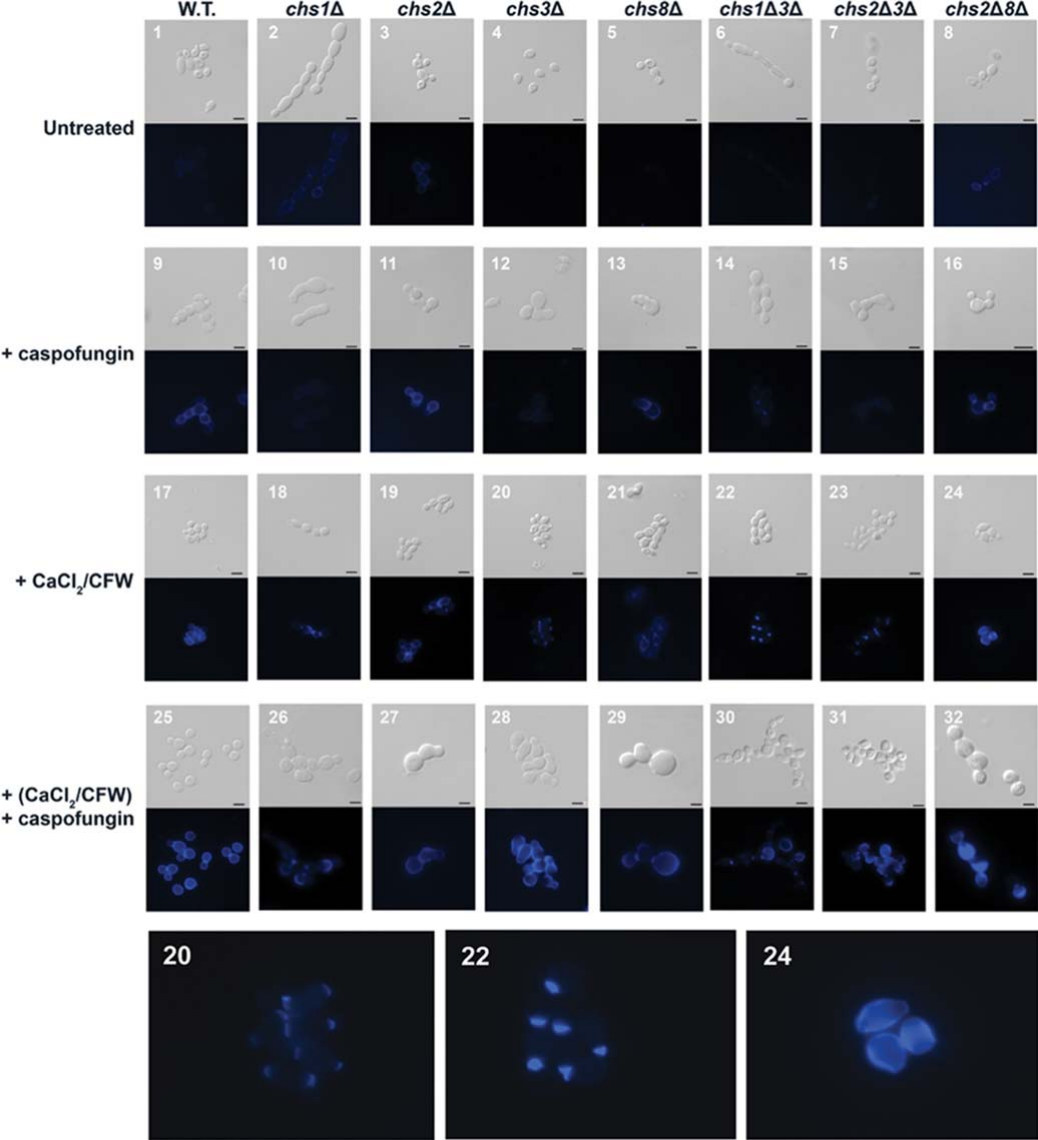
The role of chitin synthase isoenzymes in elevation of chitin levels in response to caspofungin. DIC (top panels) and fluorescent images (bottom panels) of wild type and *chs*Δ mutant strains grown in the presence and absence of 0.032 µg/ml caspofungin with and without pre-treatment of the inoculum with CaCl_2_ and CFW. Scale bars are 2 µm. Enlarged images are presented on the bottom panel showing the chitin distribution in cells induced by CaCl_2_ and CFW in the *chs3*Δ (20) and *chs1*Δ*chs3*Δ (22) mutants, with the latter showing induced synthesis of salvage septa. In (23) increased chitin formation is shown as induced by CaCl_2_ and CFW and subsequent culture in caspofungin.

**Figure 8:**
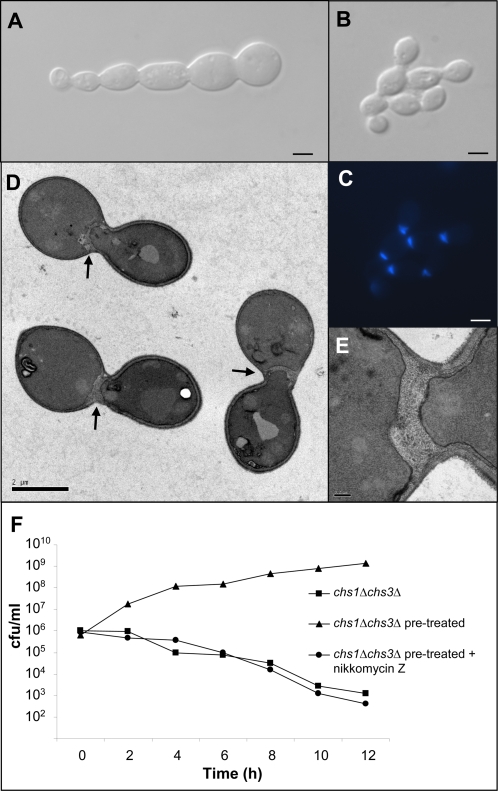
The salvage septum of *C. albicans* in the *chs1*Δ*chs3*Δ double mutant (under repressing conditions for the conditional *MRP1p-CHS1* mutant). (A, B) The chained septum-less phenotype of the *chs1*Δ*chs3*Δ mutant (A) is abrogated by pre-growth on YPD with CaCl_2_ and CFW to stimulate chitin synthesis prior to growth of the mutant cells under repressing conditions (B,C). (C) Shows CFW-fluorescence image of cells shown in (B). TEM images of proximal offset thickened septa and (D,E). Scale bars are 2 µm for (A–D) and 0.2 µm for (E). Growth of the inoculum in YPD with CaCl_2_ and CFW allows the *chs1*Δ*chs3*Δ mutant to grow in the presence of caspofungin while treatment of these cells with 10 µM nikkomycin Z leads to inhibition of growth and cell lysis (F).

**Figure 9:**
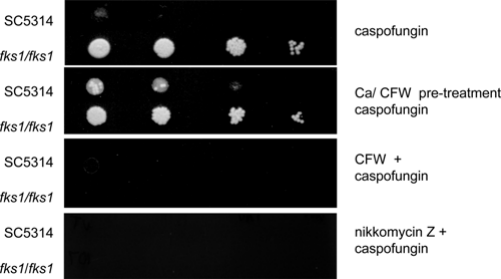
Synergistic inhibition of growth of a wild type strain (SC5314) and an *fks1* homozygous point mutant (NR3) by 16 µg/ml caspofungin and 100 µg/ml CFW or 10 µM nikkomycin Z as chitin synthase inhibitors is further demonstrated in plate sensitivity tests.

Having established that treatment with echinocandins leads to a compensatory up-regulation of chitin synthesis, we next used a panel of single and double *chs* mutants to determine which chitin synthase enzymes were required to rescue the cells from the effects of echinocandins. By measuring the pattern and relative amount of CFW fluorescence we observed that Chs3p was responsible for synthesising the majority of chitin induced by caspofungin treatment (Figure 7 panels 1–16). Pre-growing the wild-type, *chs2*Δ*chs8*Δ, *chs2*Δ and *chs8*Δ mutants in CaCl_2_ and CFW (Figure 7 panels 17, 19, 21 & 24) led to an overall increase in cell wall chitin content. Significant amounts of chitin accumulated at the poles of the *chs3*Δ and *chs2*Δ*chs3*Δ mutants when pre-grown in CaCl_2_ and CFW (Figure 7 panels 20 & 23) suggesting that pre-treatment stimulates the remaining chitin synthase enzymes to synthesize salvage chitin in the absence of Chs3p. Likewise, the *chs1*Δ and *chs1*Δ*chs3*Δ mutants had concentrated areas of chitin at the septum after pre-treatment with CaCl_2_ and CFW (Figure 7 panels 18 and 22). In all cases, pre-treatment and then exposure to caspofungin stimulated production of salvage chitin (Figure 7 panels 25–32) via activation of multiple *Chs* enzymes. Moreover, mutants lacking Chs3p and Chs1p were able to survive in the presence or absence of caspofungin when pre-grown in CaCl_2_ and CFW-containing medium (Figure 7 panels 22 and 30). This is remarkable in view of the fact that *CHS1* is essential for *C. albicans* and that Chs3p has been thus far been considered to be the key chitin synthase of the cell wall salvage pathway in *S. cerevisiae* [7]. In this double mutant, the cells grown in CaCl_2_ and CFW-containing medium had unusually bright CFW-staining and thickened septa that formed proximal to the normal location at the mother-bud neck region (Figure 7 panel 22 and Figure 8A–C). These salvage septa also stained with Wheat Germ Agglutinin (WGA)-Texas Red indicating that they were chitin rich (data not shown). Chs3p and Chs1p normally collaborate to form the chitin ring and primary septal plate of wild type septa respectively, but these salvage septa were formed in the absence of these two chitin synthases. The salvage septum was able to restore the capability for cell division, so that the formation of septum-less chains of cells and subsequent cell lysis normally associated with the lack of Chs1p was abrogated and viability was restored (Figure 8D, E and Figure 8F). Abrogation of these phenotypes associated with the *chs* mutations was entirely dependent upon chitin synthesis and could be inhibited completely by treatment with nikkomycin Z (Figure 8F). In pre-treated wild type cells or the *fks1* point mutant, inhibition of chitin assembly by CFW or chitin synthesis by nikkomycin Z was strongly synergistic with caspofungin in killing cells even under conditions that maximally induce cell wall compensatory mechanisms (Figure 9). Treatment with RO-09-3143, a selective chitin synthase inhibitor developed by Roche against the class II enzyme Chs1p [43], phenocopied all the effects of the *chs1* conditional mutation (data not shown). These observations strongly support the conclusion that the Chs2p and/or Chs8p class I chitin synthases are responsible for synthesizing the chitin in the salvage septum that rescues the cells under these conditions.

## Discussion

The echinocandins are proving to be a safe and efficacious new class of antifungal drug for the treatment of systemic mycoses [11],[44]. Laboratory-generated point mutations in the Fks1p target around the ^645^Ser hotspot region alter the affinity for these non-competitive inhibitors and results in reduced susceptibility [9],[15]. However, there are a few recorded cases of failed echinocandin therapy in the treatment of *Candida* infections caused by *C. albicans* [45]–[48], *C. glabrata* [49], *C. parapsilosis* [50],[51] and *C. krusei* [52]. In two cases, the decreased echinocandin sensitivity of recovered *C. albicans* isolates was shown to be due to mutations in *FKS1* [47],[48]. In addition, the emergence of a *C. krusei* isolate with decreased caspofungin susceptibility [53] was found subsequently to have a heterozygous mutation in the *FKS1* hotspot region [17]. It has also been suggested that an increase in cell wall chitin may explain the so-called “paradoxical effect” whereby some clinical isolates exhibit decreased sensitivity to increased concentrations of caspofungin [54]. Collectively these observations suggest that the sensitivity of a strain of *Candida* may relate in part to aspects of fungal physiology other than the affinity of the Fks1p target protein for echinocandins.

We have shown by *in vitro* experiments that *C. albicans* can rapidly respond to the presence of echinocandins by elevating chitin content, and that this response protects the cells from cell wall damage due to inhibition of β(1,3)-glucan synthesis. This may occur either by selection of a sub-population of naturally occurring chitin-rich cells, and/or by induction of the cell wall compensatory mechanisms that activate chitin synthesis. Our data predict that elevation in chitin content can offset the loss of cell wall integrity caused by echinocandin treatment. Although no direct measurements of the mechanical robustness of the cell wall have been devised in fungi, we show that survival against high levels of echinocandins is chitin synthesis-dependent and that the class I enzymes, Chs2p and Chs8p play vital roles in this respect. All treatments and conditions that led to elevation of chitin content also increased the MIC to echinocandins. It is also formally possible that changes in the cell wall, other than induction o chitin synthesis, also contribute to the changes in sensitivity to caspofungin that we have observed.

As demonstrated previously [31], at least three signal transduction pathways participate in the compensatory responses - PKC, Ca^2+^-calcineurin and HOG. Of these, the Ca^2+^-calcineurin pathway plays a key role in activating class I chitin synthases that are important for the compensatory response to caspofungin. Caspofungin treatment activated these pathways and led to increased transcription of *CHS1*, *CHS2* and *CHS8* and increased levels of Chs3p in cells.

The class II enzyme of *C. albicans*, Chs1p, is normally essential for viability and is responsible for synthesis of the primary septum and for stabilizing lateral cell wall integrity [36]. As in *S. cerevisiae*, the class IV enzyme Chs3p synthesizes the chitin ring around the rim of the primary septal plate and makes 80–90% of the total cell wall chitin of both yeast and hyphal cells [38],[40]. Remarkably, when pre-treated with CaCl_2_ and CFW, the conditional double *chs1*Δ*chs3*Δ mutant grown under restrictive conditions for *CHS1* expression was viable, had a normal morphology and was able to construct a chitin-containing septum that enabled cell division. In these cells, the only enzymes available for chitin synthesis were the two class I enzymes, Chs2p and Chs8p, which thus far have not been considered to be relevant for septum formation. Reinforcing this, nikkomycin Z which is selectively active against class I chitin synthases, prevented salvage septum synthesis and synergized strongly with caspofungin in killing the fungal cells. *C. albicans* is relatively insensitive to these inhibitors under normal conditions and a class II (*Ca*Chs1p) inhibitor has been shown to be cidal in a genetic background that lacks *Ca*Chs2p. We observed potent synergistic effects when chitin assembly and synthesis, were inhibited, even partially, by CFW and nikkomycin Z in the presence of β(1,3)-glucan synthesis inhibitors. This underlines the potential for new combination treatments, which inhibit both β(1,3)-glucan and chitin synthesis. Cidal combinations of chemotherapeutic agents can also be devised by using inhibitors of β(1,3)-glucan synthesis along with agents that block the cell wall compensatory pathways of fungi [35]. These experiments point simultaneously to the remarkable robustness and potential vulnerability of fungal cell wall biosynthesis to chemotherapeutic intervention.

## Materials and Methods

### Strains, media and growth conditions

*C. albicans* strains used in this study are listed in Table S1 provided in the Supporting Information section. *C. albicans* cultures were maintained on solid YPD medium (1% (w/v) yeast extract, 2% (w/v) mycological peptone, 2% (w/v) glucose, 2% (w/v) agar) and yeast cell cultures were grown at 30°C in YPD with shaking at 200 rpm. Hyphae were induced by growing cells in RPMI-1640 at 37°C. The MRP1p-*CHS1*/*chs1*Δ conditional mutant was maintained in medium containing maltose and grown in YPD to repress expression of *CHS1* [36].

### Antifungal agents

Cells were grown in YPD supplemented with the following antifungal agents: 0.032 µg/ml to 16 µg/ml caspofungin (Merck Research Laboratories, New Jersey, USA) dissolved in sterile water, 1.6 µg/ml cilofungin (Eli Lilly Laboratories, Indianapolis, USA) dissolved in 100% ethanol, 0.3 µg/ml echinocandin B (Eli Lilly Laboratories) dissolved in 50% (v/v) methanol, 10 µM nikkomycin Z (Bayer, Chemical Co., Leverkusen, Germany) dissolved in sterile water and 0.032 µg/ml anidulafungin (Pfizer, Sandwich, Kent) dissolved in 100% DMSO. In some experiments the inoculum was pre-treated by growing in YPD containing 0.2 M CaCl_2_ and 100 µg/ml CFW. Cultures were incubated at 30°C overnight with shaking at 200 rpm.

### Caspofungin sensitivity testing

Caspofungin was incorporated into YPD plates at 0.032 µg/ml and 16 µg/ml. In some experiments caspofungin was used in combination with 100 µg/ml CFW (Sigma-Aldrich, UK). Yeast cells were grown to late log phase in YPD and serially diluted to generate suspensions containing 1×10^6^ to 1000 cells/ml in fresh YPD. Plates were inoculated with 5 µl drops of each cell suspension and incubated for 24 h at 30°C.

### Antifungal susceptibility testing

Minimum inhibitory concentrations were determined by broth micro-dilution testing using the CLSI (formerly NCCLS) guidelines M27-A2 [55]. Drug concentrations ranged from 2 ng/ml to 16 µg/ml for caspofungin, anidulafungin and micafungin, 0.032 µg/ml to 16 µg/ml for amphotericin B, terbinafine and itraconazole and 0.13 µg/ml to 64 µg/ml for fluconazole and flucytosine. Each drug was serially diluted with sterile water in flat bottomed 96 well plates. Exponentially grown cultures were diluted and 20 µl of a 1×10^6^ culture was inoculated in either 11 ml 2× RPMI-1640 or 2× YPD and 100 µl of culture was added to each well. Plates were incubated for 24 h at 30°C for YPD plates and 37°C for RPMI-1640 plates. After incubation each well was mixed thoroughly and optical densities were read in a VERSAmax tunable microplate reader (Molecular Devices, California, USA) at 405 nm for RPMI-1640 plates and 600 nm for YPD plates.

### Measurement of CHS expression

Plasmid placpoly-6 was used for the *lacZ* promoter reporter system [56] (Uhl and Johnson, 2001). A 1 kb upstream region from the ATG start codon of each *CHS1*, *CHS2*, *CHS3* and *CHS8* ORF was cloned into the *Pst*I-*Xho*I sites of placpoly-6 generating pCHS1plac, pCHS2plac, pCHS3plac, pCHS8plac respectively as described previously [31]. *C. albicans* cultures were grown overnight on YPD at then inoculated into fresh YPD medium for 4 h, with or without echinocandins (0.032 µg/ml caspofungin and the others at the concentrations stated above). Cells were harvested after 4 h incubation, with shaking, at 30°C. Quantification of β-galactosidase activity was determined using the method previously described [31]. Specific β-galactosidase activities were expressed as nmol ο-nitrophenol produced min/mg/protein. Statistical significant differences in the assay results were determined with SPSS software using ANOVA and Post Hoc Dunnett's *T*-test, *P*<0.05. When the results displayed unequal variance the Dunnett's T3 test was applied.

### Measurement of chitin synthase activity

Mixed membrane fractions (MMF) were prepared from exponential phase yeast cells and their chitin synthase activities were measured as described previously [31].

### Measurement of cell wall chitin content

Cell walls were prepared from exponential *C. albicans* yeast cultures grown in YPD and the chitin content was measured as described previously [31].

### Western analysis of Chs3p

Overnight cultures of yeast cells of NGY477 (Chs3p-YFP) and BWP17 (untagged) were diluted 1∶100 into 50 ml YPD supplemented with uridine and 0.032 µg/ml caspofungin and incubated with shaking for 4 h at 30°C. BWP17 [57], the untagged parent strain of NGY477, provided a negative control for the anti-GFP antibody [41]. After treatment, cells were harvested by centrifugation (1 500×g, 2 min, 4°C), washed in 1 ml cold Lysis Buffer (50 mM Tris-HCl pH 7.5, 150 mM NaCl, 0.5% NP40, 2 µg/ml Leupeptin, 2 µg/ml Pepstatin, 1 mM PMSF) and finally resuspended in 250 µl cold Lysis Buffer. Cells were broken using a FastPrep machine and acid-washed glass beads. The extracts were clarified by centrifugation (16 000×g, 5 min, 4°C). Protein samples (15 µg each) were separated by SDS-polyacrylamide gel electrophoresis (SDS-PAGE) using the XCell *SureLock*™ Mini-Cell system with NuPAGE®Novex Bis-Tris 4-12% pre-cast gels in NuPAGE® MOPS-SDS Running Buffer containing NuPAGE® Antioxidant (Invitrogen Ltd, Paisley, UK) as per the manufacturer's instructions. The proteins were transferred to Invitrolon™ PVDF Membranes (Invitrogen) following the manufacturer's instructions. The membranes were then rinsed in PBS, blocked in PBS-T+10% BSA (PBS, 0.1% Tween-20, 10% (w/v) BSA) for 30 min at RT and incubated overnight at 4°C in PBS-T+5% (w/v) BSA (PBS, 0.1% Tween-20, 5% (w/v) BSA) containing a 1∶2000 dilution of anti-GFP Antibody (Roche, Basel, Switzerland). The membranes were washed five times for 5 min in PBS-T (PBS, 0.1% Tween-20) and then incubated for 1 h at RT in PBS-T+5% (w/v) BSA containing a 1∶4000 dilution of anti-mouse IgG, (Fab specific) peroxidase conjugate Antibody (Sigma-Aldrich). The membranes were washed three times for 5 min in PBS-T and the signal was detected using LumiGLO™ Reagent and Peroxide (Cell Signaling Technology, Massachusetts, USA) as per the manufacturer's instructions.

### Western analysis of Mkc1p

Western blots were carried out as above with the following modification; a 1∶1000 dilution of Phospho-p44/42 Map Kinase (Thr202/Tyr204) Antibody (Cell Signaling Technology) was used as the primary antibody. The secondary antibody was Anti-rabbit IgG, HRP-linked Antibody (Cell Signaling Technology) diluted 1∶2000.

### Fluorescence Microscopy

Samples were fixed in 10% (v/v) neutral buffered formalin (Sigma-Aldrich) and examined by phase differential interference contrast (DIC) microscopy. Cells were stained with 25 µg/ml CFW to visualize chitin. Nuclei were stained by overlaying samples with mounting media containing 1.5 µg/ml DAPI (Vector Laboratories, Peterborough, UK). Cell membrane integrity was determined by staining cells with 2 µg/ml propidium iodide (Sigma-Aldrich). All samples were examined by DIC and fluorescence microscopy using a Zeiss Axioplan 2 microscope. Images were recorded digitally using the Openlab system (Openlab v 4.04, Improvision, Coventry, UK) using a Hamamatsu C4742- 95 digital camera (Hamamatsu Photonics, Hamamatsu, Japan). CFW fluorescence was quantified for individual yeast cells using region of interest measurements. Mean fluorescence intensities were then calculated for at least 35 individual cells. In some experiments the exposure time for a series of fluorescence images was fixed so the intensity of fluorescence relative to a control of known chitin content was shown.

### Electron Microscopy

Yeast cultures were harvested by centrifugation and the pellets were fixed in 2.5% (v/v) glutaraldehyde in 0.1 M sodium phosphate buffer (pH 7.3) for 24 h at 4°C. Samples were encapsulated in 3% (w/v) low melting point agarose prior to processing to Spurr resin following a 24 h schedule on a Lynx tissue processor (secondary 1% OsO_4_ fixation, 1% Uranyl acetate contrasting, ethanol dehydration and infiltration with acetone/Spurr resin). Additional infiltration was provided under vacuum at 60°C before embedding in TAAB capsules and polymerising at 60°C for 48 h. 0.5 µm semi-thin survey sections were stained with toluidine blue to identify areas of best cell density. Ultrathin sections (60 nm) were prepared using a Diatome diamond knife on a Leica UC6 ultramicrotome, and stained with uranyl acetate and lead citrate for examination with a Philips CM10 transmission microscope (FEI UK Ltd, Cambridge, UK) and imaging with a Gatan Bioscan 792 (Gatan UK, Abingdon, UK).

### Genes used in the study

Gene nomenclature is defined at the Candida genome database (http://www.candidagenome.org/) and NCBI http://www.ncbi.nlm.nih.gov/sites/entrez).

*CHS1* (orf19.5188, XM_711849); *CHS2* (orf19.7298, XM_711340); *CHS3* (orf19.4937, XM_712573); *CHS8* (orf19.5384, XM_712667); *FKS1/GSC1* (orf19.2929, XM_716336); *MKC1* (orf19.7523, X76708); *HOG1* (orf19.895, XM_715923); *CNA1* (orf19.6033).

## Supporting Information

Figure S1 Population CFW fluorescence (chitin) heterogeneity for wild type (WT) cells treated with 0.032 µg/ml caspofungin and or 200 mM CaCl with 100 µg/m CFW (C&C). Cells were first grown for 16 h in YPD in the absence of supplements, then grown in YPD for 6 h at 30°C in the presence of caspofungin and, or CaCl + CFW. The cells were then washed in water and stained with 25 µg/ml CFW, and the relative fluorescence determined as described in the Methods. The fluorescence of fifty cells per treatment was then determined.
(0.69 MB DOC)

Table S1 *C. albicans* strains used in this study
(0.15 MB DOC)

Table S2 Fold induction of LacZ expression upon caspofungin exposure
(0.04 MB DOC)
