# Supplementary material for: Stimulation of Chitin Synthesis Rescues Candida albicans from Echinocandins
Source: PLoS Pathog. 2008 Apr 4;4(4):e1000040. doi: 10.1371/journal.ppat.1000040 (PMC2271054; doi:10.1371/journal.ppat.1000040)
Supplement: Figure S1 — Population CFW fluorescence (chitin) heterogeneity for wild type (WT) cells treated with 0.032 µg/ml caspofungin and or 200 mM CaCl with 100 µg/m CFW (C&C). Cells were first grown for 16 h in YPD in the absence of supplements, then grown in YPD for 6 h at 30°C in the presence of caspofungin and, or CaCl + CFW. The cells were then washed in water and stained with 25 µg/ml CFW, and the relative fluorescence determined as described in the Methods. The fluorescence of fifty cells per treatment was then determined. (0.69 MB DOC) [file ppat.1000040.s001.doc]

**Supplementary data: Walker et al**

Figure 1. Population CFW fluorescence (chitin) heterogeneity for wild type (WT) cells treated with 0.032 µg/ml caspofungin and or 200 mM CaCl with 100 µg/m CFW (C&C). Cells were first grown for 16 h in YPD in the absence of supplements, then grown in YPD for 6 h at 30ºC in the presence of caspofungin and, or CaCl + CFW. The cells were then washed in water and stained with 25 µg/ml CFW, and the relative fluorescence determined as described in the Methods. The fluorescence of fifty cells per treatment was then determined.
