# Supplementary material for: Stimulation of Chitin Synthesis Rescues Candida albicans from Echinocandins
Source: PLoS Pathog. 2008 Apr 4;4(4):e1000040. doi: 10.1371/journal.ppat.1000040 (PMC2271054; doi:10.1371/journal.ppat.1000040)
Supplement: Table S1 — C. albicans strains used in this study (0.15 MB DOC) [file ppat.1000040.s002.doc]

| Table S1 *C. albicans* strains used in this study | | |  |
| --- | --- | --- | --- |
| Strain | Parental strain | Genotype* | Source or Reference |
| CAF2-1 | SC5314 | *URA3/ura3*Δ::*λimm434* | [58] |
| CAI-4 | CAF2-1 | *ura3*Δ::*λimm434*/*ura3*Δ::*λimm434* | [58] |
| NGY210 | CAI-4 | *RPS1*/*rps1*Δ::pCHS1plac | [31] |
| NGY211 | CAI-4 | *RPS1*/*rps1*Δ::pCHS2plac | [31] |
| NGY212 | CAI-4 | *RPS1*/*rps1*Δ::pCHS3plac | [31] |
| NGY213 | CAI-4 | *RPS1*/*rps1*Δ::pCHS8plac | [31] |
| C155 | C154 | *chs2*Δ:: *hisG/chs2*Δ::*hisG* | [59] |
| Myco3 | Myco4 | *chs3*Δ:: *hisG/ chs3*Δ::*hisG* | [40] |
| NGY128 | CAI-4 | *chs8*Δ::*hisG/chs8*Δ::*hisG* | [39] |
| C157 | C155 | *chs2*Δ:: *hisG/chs2*Δ::*hisG;*  *chs3*Δ:: *hisG/chs3*Δ::*hisG* | [59] |
| NGY138 | CAI-4 | *chs2*Δ::*hisG/chs2*Δ::*hisG;*  *chs8*Δ:: *hisG/chs8*Δ::*hisG* | [39] |
| KWC352 | SGY243 | *chs1*Δ*::hisG/chs1*Δ*; psk-URA3-MRP1p-CHS1* | [36] |
| KWC359 | SGY243 | *chs1*Δ*::hisG/chs1*Δ*; psk-URA3-MRP1p-CHS1; chs3*Δ*/chs3*Δ | [36] |
| CM1613c | CAI-4 | *mkc1*Δ::*hisG/mkc1*Δ::*hisG* | [60] |
| DSY2101 | CAI-4 | *cna1*Δ:*:hisG/cna1*Δ:*:hisG* | [32] |
| CNC15 | RIM1000 | *hog1*Δ::*hisG*/*hog1*Δ::*hisG* | [61] |
| NR4 |  | *fks1/FKS1* | [9] |
| NR3 |  | *fks1/fks1* | [9] |
|  |  |  |  |
|  |  |  |  |

| Table S1 cont. *C. albicans* strains used in this study | | | |
| --- | --- | --- | --- |
| Strain | Parental strain | Genotype | Source or Reference |
| NGY294 | DSY2101 | *cna1*Δ::*hisG/cna1*Δ:*:hisG; RPS1/rps1*Δ::pCHS1plac | [31] |
| NGY295 | DSY2101 | *cna1*Δ::*hisG/cna1*Δ:*:hisG; RPS1/rps1*Δ::pCHS2plac | [31] |
| NGY296 | DSY2101 | *cna1*Δ::*hisG/cna1*Δ:*:hisG; RPS1/rps1*Δ::pCHS3plac | [31] |
| NGY297 | DSY2101 | *cna1*Δ::*hisG/cna1*Δ:*:hisG; RPS1/rps1*Δ::pCHS4plac | [31] |
| NGY282 | *mkc1*Δ | *mkc1*Δ::*hisG*/*mkc1*Δ::*hisG*; *RPS1*/*rps1*Δ::pCHS1plac | [31] |
| NGY283 | *mkc1*Δ | *mkc1*Δ::*hisG*/*mkc1*Δ::*hisG*; *RPS1*/*rps1*Δ::pCHS2plac | [31] |
| NGY284 | *mkc1*Δ | *mkc1*Δ::*hisG*/*mkc1*Δ::*hisG*; *RPS1*/*rps1*Δ::pCHS3plac | [31] |
| NGY285 | *mkc1*Δ | *mkc1*Δ::*hisG*/*mkc1*Δ::*hisG*; *RPS1*/*rps1*Δ::pCHS8plac | [31] |
| NGY321 | *hog1*Δ | *hog1*Δ::*hisG*/*hog1*Δ::*hisG*; *RPS1*/*rps1*Δ::pCHS1plac | [31] |
| NGY322 | *hog1*Δ | *hog1*Δ::*hisG*/*hog1*Δ::*hisG*; *RPS1*/*rps1*Δ::pCHS2plac | [31] |
| NGY323 | *hog1*Δ | *hog1*Δ::*hisG*/*hog1*Δ::*hisG*; *RPS1*/*rps1*Δ::pCHS3plac | [31] |
| NGY324 | *hog1*Δ | *hog1*Δ::*hisG*/*hog1*Δ::*hisG*; *RPS1*/*rps1*Δ::pCHS8plac | [31] |
| BWP17 | RM1000 | *his1*Δ::*hisG*/*his1*Δ::*hisG*, *arg4*Δ::*hisG*/*arg4*Δ::*hisG* | [57] |
| NGY477 | BWP17 | *his1*Δ::*hisG*/*his1*Δ::*hisG*, *arg4*Δ::*hisG*/*arg4*Δ::*hisG*, | [41] |
|  |  | *CHS3*/*CHS3*::YFP-*URA3*, *RPS1*::CIp30 |  |

*all strains apart from CAF2-1 are also *ura3*Δ::*λimm434*/*ura3*Δ::*λimm434*
