# Supplementary material for: Stimulation of Chitin Synthesis Rescues Candida albicans from Echinocandins
Source: PLoS Pathog. 2008 Apr 4;4(4):e1000040. doi: 10.1371/journal.ppat.1000040 (PMC2271054; doi:10.1371/journal.ppat.1000040)
Supplement: Table S2 — Fold induction of LacZ expression upon caspofungin exposure (0.04 MB DOC) [file ppat.1000040.s003.doc]

**Table S2: Fold induction of LacZ expression upon caspofungin exposure**

*cna1*

*hog1*

*mkc1*

WT

CHS1p-lacz

1.16

1.37

1.69

1.74

CHS2p-lacz

2.22

1.13

1.62

2.86

CHS3p-lacz

1.62

1.64

1.67

1.51

CHS8p-lacz

2.57

1.06

2.60

3.34
